# An iterative identification procedure for dynamic modeling of biochemical networks

**DOI:** 10.1186/1752-0509-4-11

**Published:** 2010-02-17

**Authors:** Eva Balsa-Canto, Antonio A Alonso, Julio R Banga

**Affiliations:** 1 Bioprocess Engineering Group, Spanish National Research Council, IIM-CSIC, 36208, Vigo-Spain

## Abstract

**Background:**

Mathematical models provide abstract representations of the information gained from experimental observations on the structure and function of a particular biological system. Conferring a predictive character on a given mathematical formulation often relies on determining a number of non-measurable parameters that largely condition the model's response. These parameters can be identified by fitting the model to experimental data. However, this fit can only be accomplished when identifiability can be guaranteed.

**Results:**

We propose a novel iterative identification procedure for detecting and dealing with the lack of identifiability. The procedure involves the following steps: 1) performing a structural identifiability analysis to detect identifiable parameters; 2) globally ranking the parameters to assist in the selection of the most relevant parameters; 3) calibrating the model using global optimization methods; 4) conducting a practical identifiability analysis consisting of two (*a priori *and *a posteriori*) phases aimed at evaluating the quality of given experimental designs and of the parameter estimates, respectively and 5) optimal experimental design so as to compute the scheme of experiments that maximizes the quality and quantity of information for fitting the model.

**Conclusions:**

The presented procedure was used to iteratively identify a mathematical model that describes the NF-*κ*B regulatory module involving several unknown parameters. We demonstrated the lack of identifiability of the model under typical experimental conditions and computed optimal dynamic experiments that largely improved identifiability properties.

## Background

Biological systems are mainly composed of genes that encode the molecular machines that execute the functions of life and networks of regulatory interactions specifying how genes are expressed, with both operating on multiple, hierarchical levels of organization [1]. Systems biology aims at understanding how such systems are organized by combining experimental data with mathematical modeling and computer-aided analysis techniques [1,2].

The modeling and simulation of biochemical networks (e.g. metabolic or signaling pathways) has recently received a great deal of attention [3-5]. The modeling framework selected depends both on the properties of the studied system and the modeling goals. Lauffenburger et al. [4,6] organized the models in terms of three main groups, depending on their level of detail: deterministic, probabilistic and statistical.

Currently, the most typical approach to representing biochemical networks is through a set of coupled deterministic ordinary differential equations intended to describe the network and the production and consumption rates for the individual species involved in the network [7]. The conceptual framework selected for the construction of rate equations enables models to be further classified as generalized mass-action-based models and power-law models [8].

Unfortunately, with model details come parameters, and most parameters are generally unknown, thereby hampering the possibility for obtaining quantitative predictions. Modern experimental techniques, such as time-resolved fluorescence spectroscopy or mass-spectrometry-based techniques, can be used to obtain time-series data for the biological system under consideration. The goal of model identification is then to estimate the non-measurable parameters so as to reproduce, insofar as is possible, the experimental data. Although apparently simple, non-linear model identification is usually a very challenging task, due to the usual lack of identifiability, either practical or, in the worst case, structural. In fact, several authors have reported difficulties in assessing unique and meaningful values for the parameters from given sets of experimental data since broad ranges of parameter values result in similar model predictions (see for example, [9-12]).

This problem has motivated the development of iterative procedures for model identification, such as those proposed by Feng and Rabitz [13], who, using a closed-loop strategy, attempted to estimate how to stimulate and how to observe a system for identification purposes. Kremling et al. [14] and Gadkar et al. [15] suggested alternative identification procedures that involve some type of experimental design, to either calculate stimuli profiles or to select species whose concentration measurements would maximally benefit model calibration and/or model discrimination.

It is important to note, however, that, in most cases, only a limited number of components in the network can be measured, usually far fewer components than incorporated in the model; only specific stimuli are available, and the system may only be stimulated in very specific ways (for example, via sustained or pulse-wise stimulation); the number of sampling times is usually rather limited, and finally, the experimental data are subject to substantial experimental noise. These constraints, together with the dynamic and typically non-linear character of the models under consideration result in identifiability problems, i.e. in the impossibility of providing a unique solution for the parameters.

Our research describes a novel general iterative identification procedure, extending the one originally outlined in Balsa-Canto et al. [16], that addresses model identification under these typical constraints and which aims to reduce the effects of the lack of identifiability.

With this aim in mind, the iterative identification procedure presented here involves the following steps:

• Analysis of structural identifiability. This step, which is often disregarded, evaluates whether the parameters may be assigned unique values from a given pair model and observables, under ideal experimental conditions, and assesses - when this is possible - the reformulation of a given model or the implementation of an iterative procedure for model calibration.

• Global ranking of parameters. This step helps decide which parameters are the most relevant to model output. In the case of lack of structural identifiability, global ranking may be used to make decisions as to reformulate the model or which parameters to estimate.

• Model calibration using global optimization methods. The model calibration problem can be formulated as a non-linear optimization problem. Unfortunately, since it is usually the case that several sub-optimal solutions are possible, the use of global optimization methods is necessary to somehow guarantee that the best possible solution is located.

• Practical identifiability analysis. Complementary to the structural identifiability test, the practical identifiability analysis enables an evaluation of the possibility of assigning unique values to the parameters from a given set of experimental data or experimental scheme, subject to experimental noise. In this paper we distinguish between two types of practical identifiability analyses: firstly, the expected quality of a given experimental scheme is analyzed *a priori *using what we call the expected uncertainty of the parameters; and secondly, the quality of the parameter estimates for a given set of experimental data using robust confidence intervals is analyzed *a posteriori*.

• Optimal experimental design via dynamic optimization. The purpose of this step is to design dynamic experiments with the aim of maximizing data quality and quantity (as measured by the Fisher information matrix) for the purpose of model calibration.

To illustrate the difficulties that may be faced when identifying a nonlinear dynamic biological model and the performance of the proposed identification procedure we consider the mathematical model that describes the NF-*κ*B regulatory module proposed by Lipniacki et al. [9].

## Methods

### Model building

**Figure 1:**
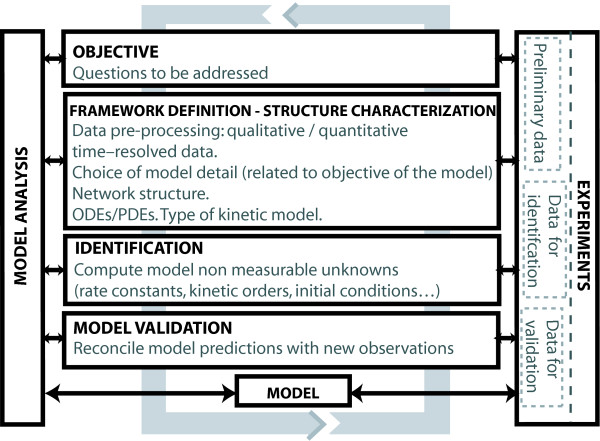
**Model building loop**.

A mathematical model has three important functions: first, it helps to better understand the biological phenomenon studied; secondly, it enables experiments to be specifically designed to make predictions of certain characteristics of the biological system that can then be experimentally verified; and finally, it summarizes the current body of knowledge in a format that can be easily communicated. Devising such a model involves a number of steps (Figure 1), commencing with a definition of its purpose and finishing with a preliminary working model.

The purpose of the model will condition the selection of the modeling framework and the information that should be included in the model. Only elements that might have an impact on the questions to be addressed by the model should be included. In this regard, account should be taken of the fact that reaction models can only include a small subset of all reactions taking place within a cell. Thus, assumptions must be made about the extent to which the species included in the model evolve independently of the species excluded from the model, and also about the species that are crucial for the purpose of the model. At this stage it is possible to define the network architecture and decide which type of modeling framework may be the most appropriate (deterministic generalized mass action based models, power-law models, stochastic models, partial differential equations, etc.)

In the next step, an initial mathematical model structure (or battery of model structures) is proposed. New experimental information must then be used to verify hypotheses, and to discriminate, if possible, among different model alternatives. The candidates will often depend on a number of unknown non-measurable parameters that can be computed by means of experimental data fitting (identification).

This crucial step provides the mathematical structure with the capacity to reproduce a given data set, make predictions and discriminate among different model candidates.

The last step is validation, which essentially means reconciling model predictions with any new data observed. This process is likely to reveal defects, in which case a new model structure and/or new (optimal) experiment is planned and implemented. This process is repeated iteratively until validation is considered to be complete and satisfactory.

Note that the success of this model-building loop relies on being able to perform experiments under a sufficient number of conditions to extract a rich ensemble of dynamic responses, to accurately measure such responses and to iterate in order to improve the predictive capabilities of the model without a significant cost.

Since model identification is a task that consumes large amounts of experimental data, an iterative identification procedure is proposed which is intended to accurately compute model unknowns while reducing experimental cost.

### Optimal identification procedure

**Figure 2:**
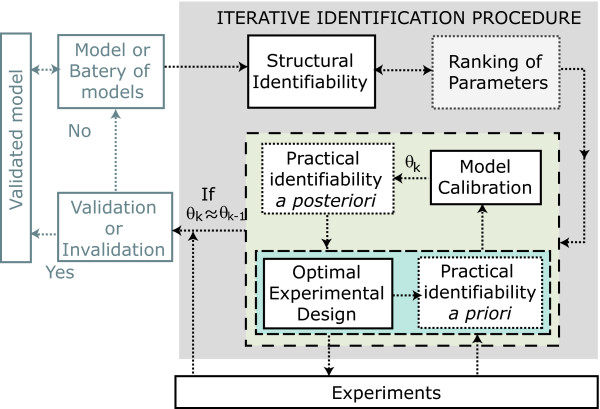
**Model building procedure incorporating the proposed model identification scheme**.

The proposed iterative identification procedure is depicted in Figure 2.

If there are several model candidates two extra steps should be included in the loop, one to analyze structural distinguishability among candidates and the other to design experiments for model discrimination [17].

#### Mathematical model formulation

We will assume a biological system described by the vector of state variables **x**(*t*) ∈ *X *⊂ , which is the unique solution of the set of nonlinear ordinary differential equations:(1)

where  corresponds to the external factors and ***θ ***∈ Θ ⊂  is the vector of model parameters where Θ is the feasible parameter space.

Moreover, given an experimental scheme, with *n*_*e *_experiments,  observables per experiment *e *and  sampling times per experiment *e *and observable *o*, **y**^*e*, *o *^∈ *Y *⊂  will regard the vector of  discrete time measurements, as follows:(2)

where  regards the *s*^*th *^sampling time for observable *o *in experiment *e*. Thus every experimental (measured) data will be denoted as  and similarly, the corresponding model predictions will be denoted as .

#### Structural identifiability analysis

Once the structure of the state-space representation, Eqns. (1)-(3), has been established, the structural identifiability problem is concerned with the possibility of calculating a unique solution for the parameters while assuming perfect data (noise-free and continuous in time and space). Structural identifiability is thus related to the model structure and possibly to the type of stimulation and independent of the parameter values.

There are, at least, two obvious reasons to asses structural identifiability: first, the model parameters have a biological meaning, and we are interested in knowing whether it is at all possible to determine their values from experimental data; second, is related with the problems that a numerical optimization approach may find when trying to solve an unidentifiable model.

There are a few methods for testing the structural identifiability of nonlinear models [18,19]: the similarity transformation approach [20], differential algebra methods [21,22] and power series approaches [23,24]. Unfortunately there is no method amenable to every model, thus at some point we have to face the selection of one of the possibilities. All of them present limitations related to the non-linearity and the size of the system under consideration, meaning by size the number of state variables, the number of parameters and the number of observables. Probably the most easy to apply, provided one uses a symbolic manipulation software, are the power series expansions methods. In this regard two possibilities have been developed: the Taylor series and the generating series.

Details of the **Taylor series approach **can be found in [23]. The approach is based on the fact that observations are unique analytic functions of time and so all their derivatives with respect to time should also be unique. It is thus possible to represent the observables by the corresponding Maclaurin series expansion and it is the uniqueness of this representation that will guarantee the structural identifiability of the system. The idea is to establish a system of non-linear algebraic equations on the parameters, based on the calculation of the Taylor series coefficients, and to check whether the system has a unique solution. The **generating series approach **[24] allows to extend the analysis to the entire class of bounded and measurable stimuli. In this case the series is generated with respect to the stimuli domain. The method requires the model to be linear in the stimuli as follows:(3)

The observables can be expanded in series with respect to time and stimuli in such a way that the coefficients of this series are **g**(**x**, ***θ***, *t *= 0) and the Lie derivatives:(5)

where *L*_**fg **_is the Lie derivative of **g **along the vector field **f**, given by:(6)

with *f*_*j *_the jth component of **f**.

If **s**(***θ***) regards the vector of all the coefficients of the series, a sufficient condition for the model to be identifiable is that there exists a unique solution for ***θ ***from **s**(***θ***), similarly to the Taylor series method. Note also that power series approaches assume that all the information on the progress of the observables is contained in the germ, i.e. the infinite set of power series coefficients evaluated at *t *= 0^+^. If the derivatives are zero, then the germ is said not to be informative and no conclusions about identifiability can be directly drawn. Later observations (*t *> 0) could give more information and restrict the set of feasible values of ***θ***. Probably the major drawback of the power series approaches is that the necessary number of power series coefficients is usually unknown. For the Taylor series approach an upper limit has been shown for bilinear and polynomial systems [25,26]. Additionally Margaria et al. (2001) [27] showed that for the combination of the Taylor series and the differential algebra approaches, *n*_*x *_+ 1 derivatives are sufficient for the case of rational systems with one observable. However there are not bounds for a general non-linear system. In addition, solving the non-linear system of equations resulting from the power series approaches is usually not a trivial task, particularly when the number of parameters is large and the number of observables is reduced. We therefore propose using the following identifiability tableaus to easily visualize the possible structural identifiability problems.

**Figure 3:**
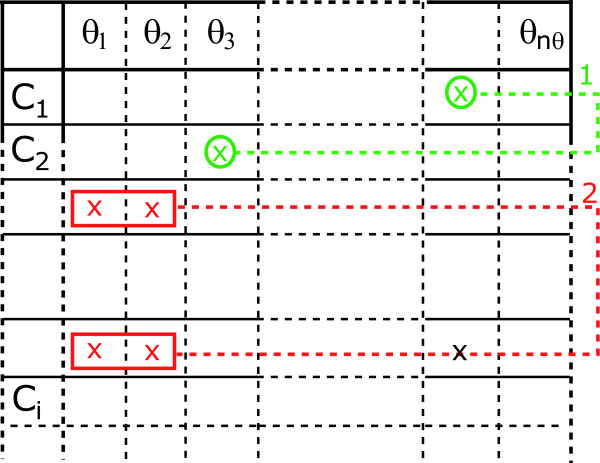
**Minimum identifiability tableau for the generating series method**. A cross in the coordinates (*i*, *j*) indicates that the corresponding non-zero generating series coefficient depends on the parameter *θ*_*j*_. Green crosses represent those parameters that can be computed from a single equation of the system. Green circles correspond to those parameters that may be uniquely identified, i.e. only one solution exist. Red crosses represent possible identifiability problems, i.e. sets of parameters that require more than 2 equations to be identified if possible. Red boxes and arrows represent sets of equations that result in an unique solution for the parameters. Numbers represent the order in which the equations were solved.

The tableau represents the non-zero elements of the Jacobian of the series coefficients with respect to the parameters. It consists of a table with as many columns as parameters and with as many rows as non-zero series coefficients, in principle, infinite, as shown in Figure 3.

If the Jacobian is rank deficient, i.e. the tableau presents empty columns, the corresponding parameters may be unidentifiable. Note that since the number of series coefficients may be infinite, unidentiability may not be fully guaranteed unless higher order series coefficients are demonstrated to be zero.

If the rank of the Jacobian coincides with the number of parameters, then it will be possible to, at least, locally identify the parameters. In this situation a careful inspection of the tableau will help to decide on an iterative procedure for solving the system of equations, as follows:

• The number of non-zero coefficients is usually much larger than the number of parameters. In practice this means that we should select the first *n*_***θ ***_rows that guarantee the Jacobian rank condition. The tableau helps to easily detect the necessary coefficients and to generate a "minimum" tableau.

• A unique non-zero element in a given row of the minimum tableau means that the corresponding parameter is structurally identifiable. If any, the parameters in this situation can be computed as functions of the power series coefficients and can be then eliminated from the "minimum" tableau to generate a "reduced" tableau. Subsequent reductions may lead to the appearance of new unique non-zero elements and so on. Thus all possible "reduced" tableaus should be built first.

• Once no more reductions are possible, one should try to solve the remaining equations. Since it is often the case that not all remaining power series coefficients depend on all parameters, the tableau will help to decide on how to select the equations to solve for particular parameters.

• If several meaningful solutions exist for a given set of parameters, then the model is said to be locally identifiable.

If the model turns out not to be completely identifiable, identifiability may be recovered by extending the set of observables, however this may not be accessible in practice. Alternatively one may consider fixing some parameters [21] or to reformulate the model.

#### Global ranking of parameters

Observables will depend differently on different parameters and this may be used to rank the parameters in order of their relative influence on model predictions. Such influence may be quantified by the use of parametric sensitivities.

Local parametric sensitivities for a given experiment *e*, observable *o *and at a sampling time  are defined as follows:(7)

They may be numerically computed by using the direct decoupled method within a backward differentiation formulae (BDF) based approach, as implemented in e.g. ODESSA [28].

The corresponding relative sensitivities, , can be used to asses the individual local parameter influence or importance, that is to establish a ranking of parameters. Brun and Reichert (2001) [29] suggested several importance factors, that may be generalized for the case of having several observables and experiments [16].

Of course, the values of the parameters are not known *a priori*, and even when optimally computed, optimal values are subject to uncertainty depending on the type of experiments and the presence of experimental noise. Consequently, the ranking for a given value of the parameters may be of limited value. Alternatively, one may compute ranking for a sufficiently large number of parameter vectors in the feasible parameter space.

The simplest approach is to apply a Monte Carlo sampling. By sampling repeatedly from the assumed joint-probability density function of the parameters and by evaluating the sensitivities for each sample, the distribution of sensitivity values, along with the mean and other characteristics, can be estimated. This approach yields reasonable results if the number of samples is quite large, requiring a great computational effort.

An alternative that can yield more precise estimates is Latin hypercube sampling (LHS). This method selects *n*_*lhs *_different values for each of the parameters, which it does by dividing the range of each parameter into *n*_*lhs *_non-overlapping intervals on the basis of equal probability. Next, from each interval one value for the parameters is selected at random with respect to the probability density in the interval.

The *n*_*lhs *_values thus obtained for the first parameter are then paired in a random manner (equally likely combinations) with the *n*_*lhs *_values for the second and successive parameters. This method allows the overall parameter space to be explored without requiring an excessively large number of samples. The importance factors will then read:(8)

where *N*_*D *_= *n*_*lhs*_*n*_*e*_*n*_*o*_*n*_*s*_, *δ*^*msqr *^and *δ*^*mabs *^quantify how sensitive a model is to a given parameter considering *δ*^*mabs *^interactions between parameters. *δ*^*max *^and *δ*^*min *^indicate the presence of outliers and provide information about the sign. *δ*^*mean *^provides information about the sign of the averaged effect a change in a parameter has on the model output.

Ordering the parameters according to these criteria, preferably in decreasing order, results in a parameter importance ranking. This information may be useful to decide on reformulating the model or to fix the less relevant parameters to improve either structural or practical identifiability.

Note that the summations will, in general, hide the different effects from the different experiments and observables unless they are in the same order of magnitude. Similar analyses may be performed for experiments and observables, thus providing information on the parameters that are more relevant to a particular observable in a particular type of experiment.

#### Model calibration

Given the measurements, the aim of model calibration or parameter identification is to estimate some or all of the parameters ***θ ***in order to minimize the distance among data and model predictions. The maximum-likelihood principle yields an appropriate cost function to quantify such distance, which, for the case of Gaussian noise with known or constant variance, reads as the widely used weighted least-squares function:(13)

where  collects the information related to a given measurement experimental noise.

Parameter identification is then formulated as a non-linear optimization problem, where the decision variables are the parameters and the objective is to minimize *J*(***θ***) subject to the system dynamics in Eqns. (1)-(3) and also, possibly, to some algebraic constraints that define the feasible region Θ.

This problem has recently received a great deal of attention in the literature. Jaqaman and Danuser presented a guide for model calibration in the context of biological systems [30] noting that there are two major issues in minimizing 13: first, the presence of local minima and second, the lack of practical identifiability.

To deal with first difficulty several authors have proposed the use of global optimization methods [31-34], since most of the model calibration problems related to biological models have several sub-optimal solutions. Recently suggested, in addition, was the use of sequential hybrid global-local methods [35,36] to enhance efficiency, particularly for highly multimodal and large scale systems.

#### Practical identifiability analysis

As already mentioned in the introduction, practical identifiability analysis enables an evaluation of the possibility of assigning unique values to parameters from a given set of experimental data or experimental scheme subject to experimental noise. We distinguish between practical identifiability a priori, which anticipates the quality of the selected experimental scheme in terms of what we will call the expected uncertainty of the parameters, and practical identifiability *a posteriori*, which assesses the quality of the parameter estimates after model calibration in terms of the confidence region.

It is important to note that the major difference between the two analyses is that, *a priori*, we have to assume a maximum experimental error, whereas, *a posteriori*, since the experimental data are already available, the experimental error may be estimated either through experimental data manipulation (when replicates of the experiments are available) or after model calibration using the residuals (i.e. the differences among model predictions and the experimental data) [37].

Possibly the simplest approach to perform such analyses given a set of simulated (*a priori*) or real (*a posteriori*) experimental data is to draw contours of the cost *J*(***θ***) by pairs of parameters. This will help detect typical practical identifiability problems, such as strong correlation between parameters, the lack of identifiability for some parameters when the contours extend to infinity, or the presence of sub-optimal solutions.

To quantify the expected uncertainty of the parameters and/or the confidence region, we rely on a Monte Carlo-based sampling method [38-40]. The underlying idea is to simulate the possibility of performing hundreds of replicates of the same experimental scheme for a given experimental error. The model calibration problem is solved for each replicate and the cloud of solutions is recorded in a matrix. Note that, in order to avoid convergence to local solutions, an efficient global optimization method is required. The cloud of solutions is assumed to correspond to, or to be fully contained in, a hyper-ellipsoid. Principal component analysis applied to the 0.95 - 0.05 interquartile range of the cloud or matrix of solutions then provides information on hyper-ellipsoid eccentricity (correlation between parameters) and pseudo-volume (accuracy of the parameters). The analysis of the histograms of the parameter solutions provides the mean value of the parameters (***μ***) and either maximum expected uncertainty (*a priori*) or the confidence intervals (*a posteriori*) for the parameters (*C*_*θ*_). See details in [40].

The obtained expected uncertainty of the parameters will allow the different experimental designs to be compared *a priori*, i.e. without performing any experiment. The richest experiment, in terms of the quantity and quality of information, will be the one with the best compromise between pseudo-volume and eccentricity.

The confidence intervals obtained for the parameters will enable a decision to be made on the need to perform further experiments to improve the quality of the parameter estimates and, thus, the predictive capabilities of the model.

#### Optimal experimental design

A crucial aspect of experimental data is data quantity and quality. As mentioned in the previous section, a given set of data may result in practical identifiability problems. This is why data generation and modeling have to be implemented as parallel and interactive processes, thereby avoiding the generation of data that may eventually turn out to be unsuited for modeling.

In addition, the use of model-based (*in silico*) experimentation can greatly reduce the effort and cost of biological experiments, and simultaneously facilitate the understanding of complex biological systems [41-44].

The model identification loop is complemented with an optimal experimental design step. The aim is to calculate the best scheme of measurements in order to maximize the richness (quantity and quality) of the information provided by the experiments while minimizing, or at least, reducing, the experimental burden [38,40].

The richness of the experimental information may be quantified by the use of the Fisher Information Matrix (ℱ) [37,45], which for the case of Gaussian known or constant variance reads as follows:(14)

where *E *represents the expectation for a given value of the parameters ***μ ***presumably close to the optimal solution θ*.

The optimal experimental design is then formulated and solved as a general dynamic optimization problem, see details in [40], that computes the time-varying stimuli profile, sampling times, experiments duration and (possibly) initial conditions so as to maximize a scalar measure of the Fisher Information Matrix subject to the system dynamics (Eqn. 1 and 3) and to other algebraic constraints associated with experimental limitations.

Regarding the selection of the scalar measure of the ℱ, several alternatives exist all of them related to the eigenvalues of the ℱ and thus related to the shape and size of the associated hyper-ellipsoid. The most popular are probably the D-optimality and E-optimality criteria, the former corresponding to the maximization of the determinant of the ℱ and the latter corresponding to the maximization of the minimum eigenvalue. From previous studies [40] it may be concluded that the E-optimality criterion offers the best quantity-quality compromise for the information, particularly for cases where the parameters are highly correlated or the sensitivities with respect to the parameters are highly uneven; otherwise D-optimality may be more successful.

## Results and Discussion

### The NF-*κ*B regulatory module

NF-*κ*B is implicated in several common diseases, especially those with inflammatory or auto immune components, such as septic shock, cancer, arthritis, diabetes and atherosclerosis [46]. Mathematical models connected to experimental data have played a key role in revealing forms of regulation of NF-*κ*B signaling and the underlying molecular mechanisms. Commencing with the original model proposed by Hoffmann et al. [47], several models have been proposed that include additional feedback loops, cross-talk with other pathways and NF-*κ*B oscillations, as detailed in the recent reviews by Lipniacki and Kimmel, [48] and Cheong et al., [49].

The model considered in this work was proposed by Lipniacki et al. [9]. This model presents several modifications with respect to the original by Hoffmann et al. [47]. Basically, while the original model accounts for the interplay among three isoforms of the inhibitory proteins I*κ*B*α*, I*κ*B*β *and I*κ*Bϵ, Lipniacki et al. consider the inhibitory roles of I*κ*B*α *and A20, incorporate new assumptions about the IKK activation and introduce the nuclear-cytoplasmic volume ratio.

The model involves two compartment kinetics of the activators IKK and NF-*κ*B, the inhibitors A20 and I*κ*B*α *and their complexes. It is assumed that IKK exists in any one of three forms: neutral (IKKn), active (IKKa) or inactive (IKKi). In the presence of an extracellular signal such as TNF, IKK is transformed into its active (phosphorylated) form. In this form it is capable of phosphorylating I*κ*B*α*, and this leads to its degradation. In resting cells, the unphosphorylated I*κ*B*α *binds to NF-*κ*B and sequesters it in an inactive form in the cytoplasm. As a result, degradation of I*κ*B*α *releases the second activator, NF-*κ*B. The free NF-*κ*B enters the nucleus and upregulates transcription of the two inhibitors I*κ*B*α *and A20 and of a large number of other genes including the control gene cgen. The newly synthesized I*κ*B*α *again inhibits NF-*κ*B, while A20 inhibits IKK by catalyzing its transformation into another inactive form in which it is no longer capable of phosphorylating I*κ*B*α*.

**Figure 4:**
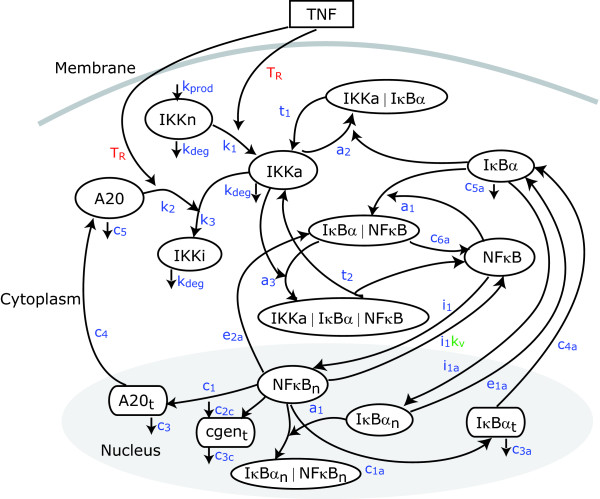
**The NF-*κ*B module**. Network model as in [9]. The notation corresponds to that used in the mathematical model. Kinetic constants are indicated in blue; *T*_*R *_regards a logical function which is 1 when the signal is activated and 0 otherwise; *k*_*v *_represents the nuclear-cytoplasmic volume ratio.

The scheme of the pathway is illustrated in Figure 4. The corresponding mathematical model consists of 15 non-linear ordinary differential equations with 30 parameters as follows [9]:

where IKKn represents the cytoplasmic concentration of neutral form of IKK kinase; IKKa, the cytoplasmic concentration of active form of IKK; IKKi, the cytoplasmic concentration of inactive IKK; I*κ*B*α*, the cytoplasmic concentration of I*κ*B*α*; I*κ*B*α*_*n*_, the nuclear concentration of I*κ*B*α*; I*κ*B*α*_*t*_, the concentration of I*κ*B*α *mRNA transcripts calculated per cytoplasmic volume V; (IKKa/I*κ*B*α*), the cytoplasmic concentration of complexes IKKa and I*κ*B*α*, equivalent notation is used for all the complexes; *T*_*R *_is a logical variable representing the presence or absence of signal; *k*_*v *_is the ratio of cytoplasmic to nuclear volumes.

## Results/Discussion

In their paper, Lipniacki et al. (2004) fixed some of the model parameters by using values from the literature. To fit the unknown parameters, they used experimental data from previous works by Lee et al. [50] and Hoffmann et al. [47]:(15)

Lipniacki et al. concluded that several different sets of parameters are capable of reproducing the data. This lack of identifiability may originate either in the structure of the model and observables selected (lack of structural identifiability) or in the type of experiments performed and the experimental noise (lack of practical identifiability). Our aim was to determine the origin of the problem and to use the model identification loop presented here to improve the quality of the parameter estimates.

### Structural identifiability analysis

To perform the analysis we take into account that Lee et al. [50] considered wild-type cells subject to a persistent TNF signal and collected data for A20 mRNA (A20_*t*_), total IKK (IKKn+IKKa+IKKi), activated IKK (IKKa), total cytoplasmic I*κ*B*α *(I*κ*B*α *+(I*κ*B*α*/NF-*κ*B)), I*κ*B*α *mRNA (I*κ*B*α*_*t*_) and free nuclear NF-*κ*B (NF-*κ*B_*n*_), and also that Hoffmann et al. [47] measured the responses of the free nuclear NF-*κ*B (NF-*κ*B_*n*_) and the cytoplasmic I*κ*B*α *(I*κ*B*α *+(I*κ*B*α*|NF-*κ*B)) in wild-type cells under persistent and pulse-wise TNF stimulation. It should be noted here that, due to the additive character of the weighted least-squares function (13) and the Fisher information matrix (14), we will regard *an experiment *as the combination of the measurements corresponding to all observables under a given stimulation even if they may not be measured simultaneously in practice.

The following is assumed:

• Only the concentrations measured by Lee et al. [50] and Hoffman et al. [47] are at our disposal.

• Initial conditions correspond to those for wild type cells after resting.

• The TNF stimulus is activated.

• Only the set ***θ ***in Eqn. are considered all the other parameters are assumed to be fixed, see details in Table 1.

**Table 1:** Nominal value for the parameters in the NF-*κ*B regulatory module

Parameter	Nominal value (θ*)	Comments
*a*_1_	0.5	Fixed
*a*_2_	0.2	Fixed
*t*_1_	0.1	To be identified
*a*_3_	1	Fixed
*t*_2_	0.1	To be identified
*c*_1*a*_	5 × 10^-7^	Fixed
*c*_2*a*_	0.0	Fixed
*c*_3*a*_	4 × 10^-4^	To be identified
*c*_4*a*_	0.5	To be identified
*c*_5*a*_	1 × 10^-4^	Fixed
*c*_6*a*_	2 × 10^-5^	Fixed
*c*_1_	5 × 10^-7^	Fixed
*c*_2_	0.0	Fixed
*c*_3_	4 × 10^-4^	Fixed
*c*_4_	0.5	Fixed
*c*_5_	3 × 10^-4^	To be identified
*k*_1_	2.5 × 10^-3^	To be identified
*k*_2_	0.1	To be identified
*k*_3_	1.5 × 10^-3^	To be identified
*k*_*prod*_	2.5 × 10^-5^	To be identified
*k*_*deg*_	1.25 × 10^-4^	To be identified
*N*_*F*_	0.06*V*	Fixed
*k*_*v*_	5	Fixed
*I*_1_	2.5 × 10^-3^	To be identified
*e*_2*a*_	0.01	To be identified
*i*_1*a*_	1 × 10^-3^	To be identified
*e*_1*a*_	5 × 10^-4^	Fixed
*c*_1*c*_	5 × 10^-7^	Fixed
*c*_2*c*_	0.0	Fixed
*c*_3*c*_	4 × 10^-4^	Fixed

The size of the model under consideration, the number of observables and the number of parameters make the application of the similarity transformation and the differential algebra approaches rather complex, thus the power series expansions will be used here.

In a first approximation to the structural identifiability problem the Taylor series approach was applied. From the analysis of the resultant tableau it is possible to asses that *i*_1_, *k*_1_, *c*_3*a *_and *i*_1*a *_are structurally identifiable. Unfortunately the complexity of the remaining equations prevents to draw clear conclusions for the rest of parameters.

**Figure 5:**
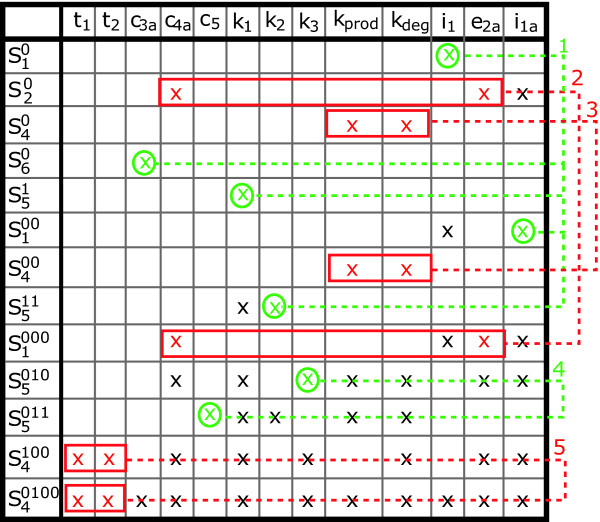
**Identifiability tableau for the NF-*κ*B model**.

The application of the generating series approach resulted, as expected, in a simpler system of equations. In fact it was possible to obtain as many coefficients as necessary to guarantee full rank Jacobian, the corresponding (full) tableau is presented in the Additional file 1: Supplemental Figure S1. Following the approach described before we obtained the minimum and the reduced tableaus (Additional file 1: Supplemental Figure S2) to demonstrate that the model is structurally identifiable (for the subset of parameters under consideration). Details are presented in the Additional file 1. Figure 5 shows a summary of the steps followed with the minimum tableau to solve the algebraic set of equations on the parameters. Since the parameters are structurally identifiable the origin of the difficulties found by Lipniacki et al. (2004) must be the lack of practical identifiability. In many practical situations this lack of identifiability originates in the lack of sensitivity of the observables with respect to the parameters. This can be assessed by performing a global sensitivity analysis and a ranking of parameters.

### Ranking of parameters

The parameters were ranked globally considering three different experimental schemes for wild-type cells. The first experiment corresponded to a persistent TNF stimulation and the second and third experiments corresponded to 1 *h *and 2 *h *pulse-wise TNF stimulations. Since it is often argued that ranking will depend on the range of parameters selected, several different tests had to be performed.

However, deciding the range of parameters is often a quite difficult task. In practice large bounds are defined so as to somehow guarantee that the real solution will lie within. Unfortunately, this approach often results in very large flat areas in the search space that make calibration extremely difficult. In addition, global analyses may lead to wrong conclusions, since the probability of considering sets of parameters that are far from the real sets increases rapidly. Whenever possible, one should use knowledge about the system to define reasonable bounds.

For this particular example we selected a reference parameter vector  taking into account the fact that the behavior of the experimental data is oscillatory under persistent TNF activation:

The reference was then used to select different bounds for the parameters. Three different tests were performed: i) within the range (), where  corresponds to the reference value of the *i*th parameter in the set ***θ***; ii) within the range () and iii) within the range (), i.e. considering that we may have underestimated, in a maximum of two, the order of magnitude of the parameters with respect to the reference. We remark that a sample of 10000 elements was used for every case.

**Figure 6:**
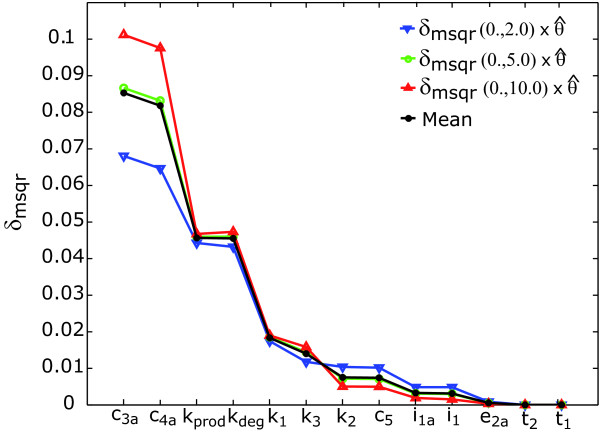
**Ranking of parameters for the NF-*κ*B example**. Parameters are ordered by decreasing *δ*_*msqr *_using the mean rank as reference.

Results obtained for all cases for the criterion *δ*_*msqr *_are presented in Figure 6 together with the mean value over all ranges. From the ranking it may be concluded that the observables are significantly sensitive to *c*_3*a*_, *c*_4*a*_, *k*_*prod *_and *k*_*deg *_and almost insensitive to *e*_2*a*_, *t*_2 _and *t*_1_, indicating possible practical identifiability problems.

In general, different ranking criteria may lead to different conclusions. In this example all criteria drive same results regarding the lack of influence of *e*_2*a*_, *t*_2 _and *t*_1 _(see Additional file 1: Supplementary Figure S3).

**Figure 7:**
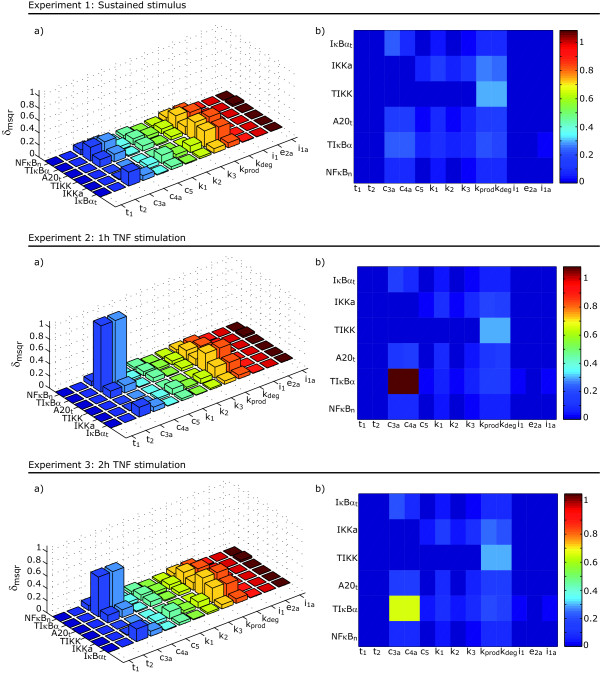
**Sensitivity analysis in the range **(). *δ*^*msqr *^measures for the different combinations of parameters and observables for the three different experiments.

As already mentioned before, the summations over experiments and observables may hide some relevant information. For example, from Figure 6 it is not possible to asses the effect of using pulse-wise stimulation or what are the parameters that are more relevant to the different observables evolution. To analyze this information we considered the sensitivities for the range () (closest to the mean behavior) in more detail. Results are depicted in Figure 7.

From the figures it may be concluded that certain observables become more sensitive to certain parameters under short pulse-wise stimulation (Experiment 2). This is the case, for example, when looking at the sensitivities with respect to *c*_3*a*_, *c*_4*a *_or *i*_1_. Note that only the measurements of total cytoplasmic I*κ*B*α *provides information about *i*_1 _and *i*_1*a *_and also the fact that we obtain almost no information about *t*_2_, *t*_1 _and *e*_2*a*_.

It is important to underline that for the case of *i*_1_, experiments under sustained stimulation appear not to be relevant whereas the model becomes more sensitive to *c*_5 _or *k*_2 _under sustained stimulation. It can thus be expected that using an experimental scheme combining a sustained stimulation experiment with (optimally designed) pulse-wise stimulation experiments will increase overall sensitivity and thus improve identifiability properties.

Taking into account the results the vector of parameters ***θ ***is partitioned into two new vectors ***θ***_*κ *_and  as follows:(16)

The components of ***θ***_*κ *_will be now considered in the next steps of the identification loop, the components in  will remain fixed to a nominal value since their presence for model calibration will be a clear source of practical identifiability problems.

### Practical identifiability analysis

To establish a basis for comparison we first consider the problem as addressed by Lipniacki et al., i.e. with all parameters in set ***θ ***and the experimental scheme available from Lee et al. [50] and Hoffmann et al. [47], to be referred to henceforth as *ES1*. The results obtained for the identifiability analysis will be considered as reference (and denoted by *REF*).

For this purpose we can perform a battery of hundreds of *in silico *experiments (1000 experiments in this research) under such experimental conditions, getting experimental data with zero-mean Gaussian noise with unknown varying variance but with a maximum corresponding to 10%.

To perform the quantitative analysis according to the Monte Carlo approach the model calibration problem was solved for all sets of data by using the recently developed global optimization method based on Scatter Search (SSm, [51]) and with bounds for the parameters of ().

**Figure 8:**
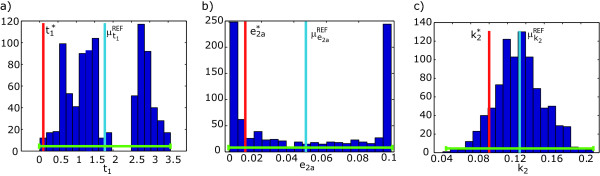
**Practical identifiability analysis for the full set *θ***. Illustrative examples of the histograms of the solutions achieved with the Monte-Carlo based approach for *t*_1_, *e*_2*a *_and *k*_2 _under the experimental scheme *ES1*.

Table 2 summarizes the results obtained confirming what was already expected from the ranking of parameters. The lack of influence of some parameters on the observables induce lack of practical identifiability. The mean value obtained for the parameters is far from the nominal. This is especially notorious for *t*_1_, *t*_2 _and *e*_2*a *_but also for *k*_2_, *k*_3_, *k*_*prod*_, *k*_*deg *_for which the relative distance is over the 20%. If we take a look at the illustrative examples of the confidence intervals in Figure 8 we may observe three different situations. Due to the lack of influence on the observables, for the case of *t*_1 _the objective function seems to be noisy and therefore the solution is hard to find even for global optimization methods and for *e*_2*a *_the objective function seems to be flat therefore the optimization method may achieve any solution in the allowed range but with a significant tendency to get trapped in the bounds. For the case *k*_2 _and all other parameters, with influence on the observables, there is one unique solution and the solver is able to find it in all runs.

**Table 2:** Practical identifiability analysis for the experimental scheme *ES1 *with () represents the nominal value for the parameters; *δ*^*REF *^is the parameter mean value computed by the Monte-Carlo based approach; *δ*^*REF *^is the relative distance between the mean and the nominal computed as ,  corresponds to the predicted maximum uncertainty of the given parameter and  represents the uncertainty with respect to *μ*^*REF *^in %.

Parameter	θ*	***μ***^***REF***^	***δ***^***REF ***^(in %)		**(in %)**
*t*_1_	0.10	1.77	1680	1.79	100.7

*t*_2_	0.10	6.16	6060	3.03	49.1

*c*_3*a*_	4.00 10^-4^	4.00 × 10^-5^	3.09	2.80 × 10^-5^	6.90

*c*_4*a*_	0.50	0.50	0.60	0.08	15.9

*c*_5_	3.00 × 10^-4^	3.07 × 10^-4^	2.49	1.02 × 10^-4^	33.1

*k*_1_	2.50 × 10^-3^	2.45 × 10^-3^	2.04	5.34 × 10^-4^	21.7

*k*_2_	0.10	0.13	33.3	0.08	60.2

*k*_3_	1.50 × 10^-3^	1.18 × 10^-3^	21.1	8.08 × 10^-4^	68.3

*k*_*prod*_	2.50 × 10^-5^	3.25 × 10^-5^	29.9	3.19 × 10^-5^	98.3

*k*_*deg*_	1.25 × 10^-4^	1.63 × 10^-4^	33.4	1.62 × 10^-4^	99.9

*i*_1_	2.50 × 10^-3^	2.40 × 10^-3^	3.85	6.38 × 10^-4^	26.5

*e*_2*a*_	0.01	4.74 × 10^-3^	374	5.30 × 10^-3^	110.9

*i*_1*a*_	1.00 × 10^-3^	9.74 × 10^-4^	0.75	2.42 × 10^-4^	24.3

Results obtained justify the fact addressed by Lipniacki et al (2004)., the origin of multiple equivalent solutions is the poor practical identifiability originated in the lack of influence of some parameters in the available observables.

If we compare the results with the ones obtained considering only the set ***θ***_*κ*_, Table 3 shows a significant improvement regarding both the ***μ ***value, the relative distance to the nominal and the expected uncertainties. The following should be remarked:

**Table 3:** Practical identifiability analysis for the experimental scheme *ES1 *with () represents the nominal value for the parameters; *μ*^*ES*1 ^is the parameter mean value computed by the Monte-Carlo based approach; *δ*^*ES*1 ^is the relative distance between the mean and the nominal computed as ,  corresponds to the predicted maximum uncertainty of the given parameter and  represents the uncertainty with respect to *μ*^*ES*1 ^in %.

Parameter	θ*	***μ***^***ES*1**^	*δ*^***ES*1**^(*in*%)		**(in %)**
*c*_3*a*_	4.00 × 10^-4^	4.00 × 10^-5^	0.02	2.20 × 10^-5^	5.40

*c*_4*a*_	0.50	0.50	0.66	0.046	9.07

*c*_5_	3.00 × 10^-4^	3.01 × 10^-4^	0.26	1.23 × 10^-4^	40.8

*k*_1_	2.50 × 10^-3^	2.49 × 10^-3^	0.46	5.01 × 10^-4^	20.1

*k*_2_	0.10	0.10	1.97	0.04	44.0

*k*_3_	1.50 × 10^-3^	1.49 × 10^-3^	0.95	5.00 × 10^-4^	33.7

*k*_*prod*_	2.50 × 10^-5^	2.60 × 10^-5^	2.90	1.40 × 10^-5^	53.7

*k*_*deg*_	1.25 × 10^-4^	1.29 × 10^-4^	3.41	7.80 × 10^-5^	60.8

*i*_1_	2.50 × 10^-3^	2.49 × 10^-3^	0.26	4.22 × 10^-4^	16.9

*i*_1*a*_	1.00 × 10^-3^	1.00 × 10^-3^	0.27	1.82 × 10^-4^	18.1

*c*_3*a *_and *c*_4*a *_can be already be appropriately estimated. The ***μ ***value is less than a 1% relative distance to the nominal ("real") value. In addition the expected uncertainties are less than a 10% which is in the order of the experimental error. As a consequence *c*_3*a *_and *c*_4*a *_can be removed from the subsequent steps in the identification procedure for the remaining parameters, denoted as , ***μ ***value is within the 5% of the nominal but the uncertainties for most of the parameters are over the 20% and over the 50% for *k*_*prod *_and *k*_*deg*_. Taking a look at the eccentricity values by pairs of parameters we will found out that in fact *k*_*prod *_and *k*_*deg *_are the most correlated pair with an eccentricity value of 14.7.

### Optimal experimental design

In order to improve the identifiability properties of  we considered a parallel-sequential optimal experimental design, in such a way that the information reported by the experimental scheme *ES1 *was taken into account by introducing the experiments in the Fisher Information Matrix (Eqn. 14). New experiments were designed within the following experimental constraints:

• Initial conditions correspond to those for wild type cells after resting.

• The TNF stimulus is activated and may be pulse-wise. In order to make the experiments more easily implementable in practice a maximum of two pulses is allowed.

• The maximum number of sampling times will be 15 and they may be optimally located.

• The experimental noise corresponds to a maximum variance of the 10%.

• The reference value for the parameters in the ℱ (Eqn. 14) corresponds to the ***μ***^*ES*1 ^(Table 3).

Regarding the ℱ based criteria for optimal experimental design, the D- and E-optimality criteria are the usually preferred ones. For this particular example, and attending to the eccentricity values corresponding to *ES1*, E-optimality seemed to be the most suitable, since this promotes the simultaneous reduction of the expected uncertainty and the eccentricity.

**Figure 9:**
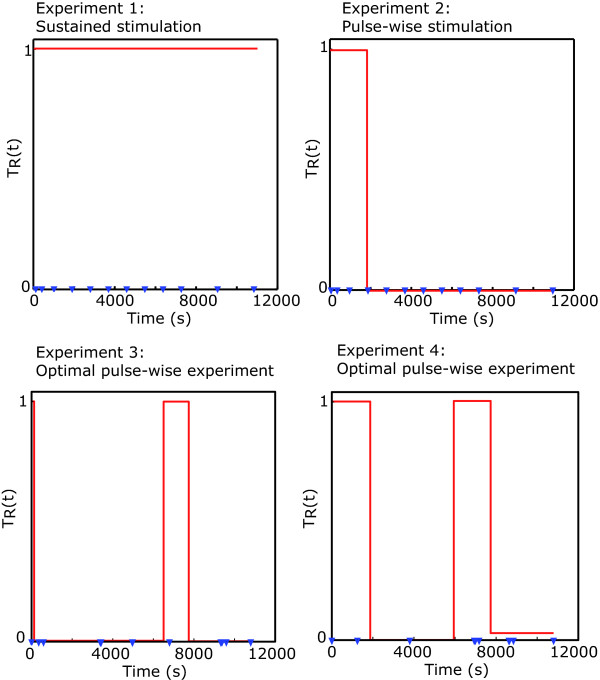
**Experiments performed throughout the identification procedure**.

The new experiment consists of performing two pulses and 15 optimally located sampling times (see Figure 9). Detailed analysis of the identifiability properties are incorporated in the Additional file 1: Supplemental Tables S1 and S2 showing how the addition of the optimally designed experiment led the mean value ***μ***^*ES*2 ^to practically coincide, less than 1% relative error, with the nominal θ* value. In addition the expected uncertainty has substantially improved as compared to the expected uncertainties found for the experimental scheme *ES1*. It should be remarked that now the worst case is of around the 32% whereas for *ES1 *it was of around 60%, in addition the maximum eccentricity, which again corresponds to the pair *k*_*prod *_- *k*_*deg*_, has been substantially reduced, to a value of 8.2.

The estimations of *k*_3_, *i*_1 _and *i*_1*a *_are now satisfactory with less than 0.5% error with respect to the nominal value and expected uncertainties of around the 10%. Next step is to compute a new optimal experimental design for the remaining parameters by using ***μ***^*ES*2 ^as a reference.

Table 4 presents a summary of the results for the overall process, revealing that the addition of a new optimally designed experiment further improved results. The maximum expected uncertainty corresponds to *c*_5 _with a value of around 17% which is quite reasonable. In addition the maximum eccentricity is now of 5.6, thus being the correlation among the parameters substantially reduced from the first experiment. Figure 9 presents the resulting set of experiments, both experiments make use of the maximum allowed number of pulses. And although the location of the pulses is rather similar in both experiments, the duration of the pulses is significantly different. It should be noted that the experiments are designed in sequence, the information from previous experiments is considered at the time of designing a new experiments, this enables the possibility of obtaining complementary information from the different experiments which reduces correlation among parameters.

**Table 4:** Summary of the practical identifiability analysis for the successive experimental schemes: a) Predicted maximum uncertainty of the given parameter in %, b) Relative distance between the mean and the nominal value of the parameters in %.

**a)**	***ES*1**			**b)**	***ES*1**		
					
*c*_3*a*_	**5.40**			*c*_3*a*_	**0.02**		
					
*c*_4*a*_	**9.07**	*ES*2	*ES*3	*c*_4*a*_	**0.66**	*ES*2	*ES*3
	
*c*_5_	40.8	32.3	**16.9**	*c*_5_	0.26	0.38	**0.6**
	
*k*_1_	20.1	18.0	**10.7**	*k*_1_	0.46	0.19	**0.18**
	
*k*_2_	44.0	14.9	**7.85**	*k*_2_	1.97	0.51	**0.25**
	
*k*_3_	33.7	**5.47**		*k*_3_	0.95	**0.10**	
	
*k*_*prod*_	53.7	23.8	**13.2**	*k*_*prod*_	2.90	0.42	**0.05**
	
*k*_*deg*_	60.8	26.3	**15.6**	*k*_*deg*_	3.41	0.44	**0.03**
	
*i*_1_	16.9	**10.4**		*i*_1_	0.26	0.12	
	
*i*_1*a*_	18.1	**8.94**		*i*_1*a*_	0.27	0.40	

Underlined values represent the worst value for the given experimental scheme. Bold face values represent the best value achieved for each parameter at the end of the identification procedure.

**Figure 10:**
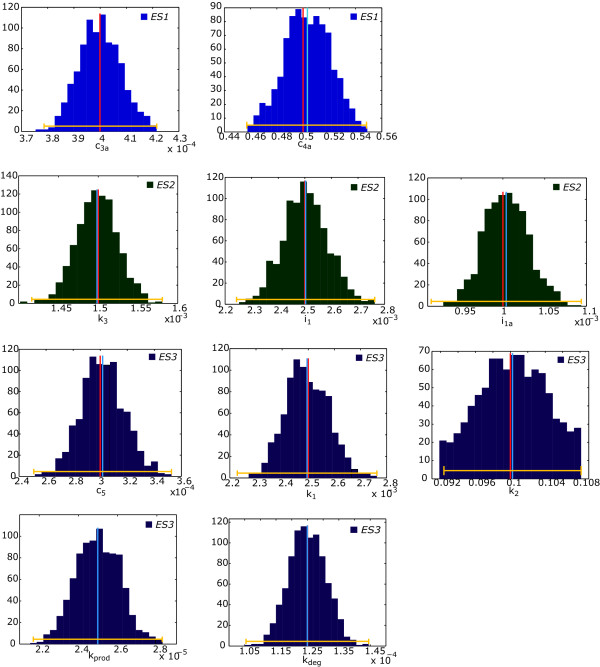
**Expected uncertainties for all parameters at the end of the identification procedure**. Red line indicates the nominal value of the parameter, blue line indicates the mean value for the given experiment and yellow line indicates the estimated expected uncertainty.

**Figure 11:**
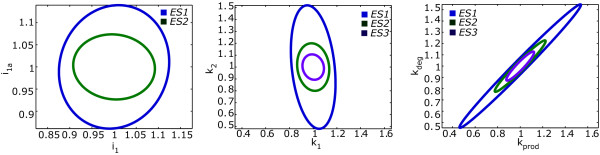
**Illustrative examples of the evolution of the robust uncertainty ellipses for several pairs of parameters**. *k*_*prod*_-*k*_*deg *_the most correlated parameters in all experimental schemes, *i*_1_-*i*_1*a *_the less correlated parameters in *ES1 *and *ES2 *and *k*_1_-*k*_2 _the less correlated parameters in *ES3*.

Figure 10 shows the evolution of the expected uncertainties for all parameters throughout the identification procedure and Figure 11 presents the comparison of the ellipses for the most and the lest correlated pairs of parameters (detailed plots of the expected uncertainties by pairs of parameters are shown in the Additional file 1: Supplemental Figures S4 and S5).

## Conclusions

It has been largely recognized that solving the solution of parameter identification problems becomes harder with the size of the problem, particularly when the ratio between the number of observables and experimental data and the number of parameters is low, since these induce multimodality and lack of structural and/or practical identifiability.

This research describes an iterative identification procedure for non-linear dynamic biological models that is intended to improve parameter identification, i.e. to reduce the dimensionality of the problem when possible and to improve identifiability properties, and therefore to avoid premature (and probably wrong) conclusions about the explanatory and predictive capabilities of a particular model. The procedure involves the following steps: structural and practical identifiability analysis, global ranking of parameters, parameter estimation using efficient global optimization techniques and optimal experimental design.

As an illustrative example, we considered parameter estimation of the model describing the NF-*κ*B module proposed by Lipniacki et al. [9]. Using the identifiability tableau based on the generating series coefficients, the possibility of simultaneously estimating the entire set of parameters was revealed. With the support of the global ranking of parameters we were able to predict the insensitivity of the observables to some of the parameters and the consequent lack of practical identifiability. After fixing such parameters we proceeded throughout the identification procedure. The practical identifiability analysis for the available experimental schemes indicated high correlation between some pairs of parameters in the subset and large expected uncertainties for the parameters. The final stage was to design two new optimal experiments that were able to substantially improve the quality of the parameter estimates. This case study clearly reveals the usefulness of the proposed identification procedure to improve efficiency and robustness during model development in systems biology.

The methodology described here has been implemented in a software toolbox, AMIGO, which is available from the authors upon request.

## Authors' contributions

EBC and JRB contributed to the conception and design of the work. EBC implemented the iterative identification procedure, performed the computations and drafted the manuscript. AAA and JRB gave valuable advises and helped to draft the manuscript. All authors read and approved the final manuscript.

## Supplementary Material

Additional file 1 **Further details on the application of the identification procedure to the mathematical model of the NF-*κ*B regulatory module**. Additional file 1 presents further details on the analysis of the structural identifiability, the ranking of parameters, the optimal experimental design and the corresponding identifiability analysis for the of NK*κ*B example. Click here for file

## References

[B1] Ideker T Galitski T Hood L A New Approach to Decoding Life: Systems Biology Annu Rev Genomics Hum Genet 2001 2 343 372 10.1146/annurev.genom.2.1.343 11701654

[B2] Kitano H Systems Biology: A Brief Overview Science 2002 295 1662 1664 10.1126/science.1069492 11872829

[B3] Cho KH Wolkenhauer O Analysis and modelling of signal transduction pathways in systems biology Biochem Soc Trans 2003 31 1503 1509 10.1042/BST0311503 14641099

[B4] Janes K Lauffenburger D A biological approach to computational models of proteomic networks Curr Op Chem Biol 2006 10 73 80 10.1016/j.cbpa.2005.12.016 16406679

[B5] Klipp E Liebermeister W Mathematical modelling of intracellular signaling pathways BMC Neuroscience 2006 7 Suppl 1:S10 17118154 10.1186/1471-2202-7-S1-S10 PMC1775040

[B6] Aldridge B Burke J Lauffenburger D Sorger P Physicochemical modelling of cell signalling pathways Nature Cell Biology 2006 8 11 1195 1203 10.1038/ncb1497 17060902

[B7] Wolkenhauer O Ullah M Kolch W Cho K Modeling and simulation of intracellular dynamics: Choosing an appropriate framework IEEE Trans on Nanobioscience 2004 3 3 200 207 10.1109/TNB.2004.833694 15473072

[B8] Vera J Balsa-Canto E Wellstead P Banga J Wolkenhauer O Power-law models of signal transduction pathways Cellular signalling 2007 19 1531 1541 10.1016/j.cellsig.2007.01.029 17399948

[B9] Lipniacki T Paszek P Brasier A Luxon B Kimmel M Mathematical model of NF*κ*B regulatory module J Theor Biol 2004 228 195 215 10.1016/j.jtbi.2004.01.001 15094015

[B10] Brown K Hill C Calero G Myers C Lee K Sethna J Cerione R The statistical mechanics of complex signaling networks:nerve growth factor signaling Phys Biol 2004 1 184 195 10.1088/1478-3967/1/3/006 16204838

[B11] Achard P Schutter ED Complex parameter landscape for a complex neuron model PLOS Computational Biology 2006 2 7 0794 0803 10.1371/journal.pcbi.0020094 PMC1513272 16848639

[B12] Piazza M Feng X Rabinoswitz J Rabitz H Diverse metabolic model parameters generate similar methionine cycle dynamics J Theor Biol 2008 251 4 628 639 10.1016/j.jtbi.2007.12.009 18313076 PMC2386584

[B13] Feng XJ Rabitz H Optimal Identification of Biochemical Reaction Networks Biophys J 2004 86 3 1270 1281 10.1016/S0006-3495(04)74201-0 14990460 PMC1303968

[B14] Kremling A Fischer S Gadkar K Doyle F Sauter T Bullinger E Allgower F Gilles E A benchmark for methods in reverse engineering and model discrimination: Problem formulation and solutions Genome Research 2004 14 9 1773 1785 10.1101/gr.1226004 15342560 PMC515324

[B15] Gadkar K Gunawan R III FD Iterative approach to model identification of biological networks BMC Bioinformatics 2005 6 155 10.1186/1471-2105-6-155 15967022 PMC1189077

[B16] Balsa-Canto E Banga JR Alonso AA Allgöwer F, Reuss M An optimal identification procedure for model development ins systems biology: Applications in Cell Signalling Foundations of Systems Biology in Engineering 2007 51 56

[B17] Agpar J Toettcher J Endy D White F Tidor B Stimulus design for model selection and validation in cell signaling PLoS Computational Biology 2008 4 2 e30 10.1371/journal.pcbi.0040030 18282085 PMC2323406

[B18] Chapman MJ Godfrey K Chappell MJ Evans ND Structural identifiability for a class of non-linear compartmental systems using linear/non-linear splitting and symbolic computation Math Biosci 2003 183 1 14 10.1016/S0025-5564(02)00223-7 12604132

[B19] Xia X Moog CH Identifiability of nonlinear systems with applications to HIV/AIDS models IEEE Trans Aut Cont 2003 48 2 330 336 10.1109/TAC.2002.808494

[B20] Vajda S Godfrey K Rabitz H Similarity transformation approach to identifiability analysis of nonlinear compartmental models Mathematical Biosciences 1989 93 217 248 10.1016/0025-5564(89)90024-2 2520030

[B21] Ljung L Glad T On global identifiability of arbitrary model parameterizations Automatica 1994 30 2 265 276 10.1016/0005-1098(94)90029-9

[B22] Bellu G Saccomani MP Audoly S D'Angiò L DAISY: A new software tool to test global identifiability of biological and physiological systems Computer Methods and Programs in Biomedicine 2007 88 52 61 10.1016/j.cmpb.2007.07.002 17707944 PMC2888537

[B23] Pohjanpalo H System identifiability based on power-series expansion of solution Math. Biosci 1978 41 1-2 21 33 10.1016/0025-5564(78)90063-9

[B24] Walter E Lecourtier Y Global approaches to identifiability testing for linear and nonlinear state space models Mathematics and Computers in Simulation 1982 24 472 482 10.1016/0378-4754(82)90645-0

[B25] Vajda S Structural identifiability of dynamical systems International Journal of Systems Science 1983 14 1229 1247 10.1080/00207728308926526

[B26] Vajda S Deterministic identifiability and algebraic invariants for polynomial systems IEEE Transactions on Automatic Control 1987 32 2 182 184 10.1109/TAC.1987.1104546

[B27] Margaria G Riccomagno E Chappell M Wynn H Differential algebra methods for the study of the structural identifiability of rational function state-space models in the biosciences Mathematical Biosciences 2001 174 1 26 10.1016/S0025-5564(01)00079-7 11595254

[B28] Leis JR Kramer MA Sensitivity Analysis of Systems of Differential and Algebraic Equations Comp & Chem Eng 1985 9 3 93 96

[B29] Brun R Reichert P Practical identifiability analysis of large environmental simulation models Water Resources Res 2001 37 1015 1030 10.1029/2000WR900350

[B30] Jaqaman K Danuser G Linking data to models: data regression Nat Rev Mol Cell Bio 2006 7 11 813 819 10.1038/nrm2030 17006434

[B31] Moles C Mendes P Banga J Parameter estimation in biochemical pathways: a comparison of global optimization methods Genome Research 2003 13 2467 2474 10.1101/gr.1262503 14559783 PMC403766

[B32] Zwolak J Tyson J Watson L Globally optimised parameters for a model of mitotic control in frog egg extracts IEE Proc Systems Biology 2005 152 2 81 92 10.1049/ip-syb:20045032 17044236

[B33] Polisetty P Voit E Gatzke E Identification of metabolic system parameters using global optimization methods Theor Biol & Med Mod 2006 3 4 10.1186/1742-4682-3-4 PMC1413512 16441881

[B34] Rodriguez-Fernandez M Egea JA Banga J Novel Metaheuristic for Parameter Estimation in Nonlinear Dynamic Biological Systems BMC Bioinformatics 2006 7 483 10.1186/1471-2105-7-483 17081289 PMC1654195

[B35] Rodriguez-Fernandez M Mendes P Banga J A hybrid approach for efficient and robust parameter estimation in biochemical pathways Biosystems 2006 83 2-3 24 10.1016/j.biosystems.2005.06.016 16236429

[B36] Balsa-Canto E Peifer M Banga J Timmer J Fleck C Hybrid optimization method with general switching strategy for parameter estimation BMC Systems Biology 2008 2 26 10.1186/1752-0509-2-26 18366722 PMC2373877

[B37] Walter E Pronzato L Identification of Parametric Models from Experimental Data 1997 Springer, Masson

[B38] Balsa-Canto E Rodriguez-Fernandez M Alonso AA Banga JR Cánovas M, Iborra J, Manjón A Computational design of optimal dynamic experiments in systems biology: a case study in cell signaling Understanding and Exploiting Systems Biology in Bioprocesses and Biomedicine 2006 Fundación CajaMurcia 103 117

[B39] Joshi M Seidel-Morgenstern A Kremling A Exploiting the bootstrap method for quantifying parameter confidence intervals in dynamical systems Metabolic Engineering 2006 8 447 455 10.1016/j.ymben.2006.04.003 16793301

[B40] Balsa-Canto E Alonso A Banga J Computational Procedures for Optimal Experimental Design in Biological Systems IET Systems Biology 2008 2 4 163 172 10.1049/iet-syb:20070069 18681746

[B41] van Riel N Dynamic modelling and analysis of biochemical networks: Mechanism-based models and model-based experiments Brief Bioinform 2006 7 4 364 374 10.1093/bib/bbl040 17107967

[B42] Kremling A Saez-Rodriguez J Systems Biology - An engineering perspective J Biotechnol 2007 129 329 351 10.1016/j.jbiotec.2007.02.009 17400319

[B43] Banga JR Balsa-Canto E Parameter estimation and optimal experimental design Essays in Biochemistry 2008 45 195 210 10.1042/BSE0450195 18793133

[B44] Kreutz C Timmer J Systems biology: experimental design FEBS J 2009 276 923 942 10.1111/j.1742-4658.2008.06843.x 19215298

[B45] Ljung L System identification: Theory for the user 1999 New Jersey: Prentice Hall

[B46] Kumar A Takada Y Boriek A Aggarwal B Nuclear Factor-*κ*B: its role in health and disease J Mol Med 2005 82 7 434 448 10.1007/s00109-004-0555-y 15175863

[B47] Hoffmann A Levchenko A Scott M Baltimore D The IkB-NF-kB signaling module: temporal control and selective gene activation Science 2002 298 1241 1245 10.1126/science.1071914 12424381

[B48] Lipniacki T Kimmel M Deterministic and Stochastic models of NF*κ*B pathway Cardiovasc Toxicol 2007 7 215 234 10.1007/s12012-007-9003-x 17943462

[B49] Cheong R Hoffmann A Levchenko A Understanding NF-*κ*B signaling via mathematical modeling Molecular Systems Biology 2008 4 192 10.1038/msb.2008.30 18463616 PMC2424295

[B50] Lee E Boone D Chai S Libby S Chien M Lodolce J Ma A Failure to regulate TNF-induced NF-*κ*B and cell death responses in A20-deficient mice Science 2000 289 2350 2354 10.1126/science.289.5488.2350 11009421 PMC3582399

[B51] Egea JA Rodriguez-Fernandez M Banga JR Marti R Scatter Search for Chemical and Bio-Process Optimization J Global Optim 2007 37 3 481 503 10.1007/s10898-006-9075-3

