# Supplementary material for: An iterative identification procedure for dynamic modeling of biochemical networks
Source: BMC Syst Biol. 2010 Feb 17;4:11. doi: 10.1186/1752-0509-4-11 (PMC2838808; doi:10.1186/1752-0509-4-11)
Supplement: Additional file 1 — Further details on the application of the identification procedure to the mathematical model of the NF-κB regulatory module. Additional file 1 presents further details on the analysis of the structural identifiability, the ranking of parameters, the optimal experimental design and the corresponding identifiability analysis for the of NKκB example. [file 1752-0509-4-11-S1.PDF]

Additional file 1

Further details on the application of the identification procedure to the mathematical model of the NF-κB regulatory module

Eva Balsa-Canto, Antonio A. Alonso and Julio R. Banga
Bioprocess Engineering Group, Spanish National Research Council, IIM-CSIC, Spain

Structural identifiability analysis

Model structural identifiability is the question whether the parameters of a given model can be uniquely, globally or locally, determined from data. As described in the main text, to check for the structural identifiability several possibilities exist. However the power series expansion approaches, and particularly the generating series method, seem to be the most amenable to the mathematical model that describes the NF-κB regulatory module. Here we present in more detail the application of the generating series approach. It should be noted that the generating series method requires the input to be analytical, this is often guaranteed in practice since at least one of the experiments will be performed under sustained stimulation. The generating series method is based on the computation of successive Lie derivatives. Since we have to consider 13 parameters, we will need at least 13 non-zero Lie derivatives. The Jacobian of the non-zero coefficients with respect to the parameters was computed and new terms added till the rank of the Jacobian was 13. The following coefficients were computed: $L_{\mathbf{f}_0}\mathbf{g}$, $L_{\mathbf{f}_1}\mathbf{g}$, $L_{\mathbf{f}_0}L_{\mathbf{f}_0}\mathbf{g}$, $L_{\mathbf{f}_0}L_{\mathbf{f}_1}\mathbf{g}$, $L_{\mathbf{f}_1}L_{\mathbf{f}_0}\mathbf{g}$, $L_{\mathbf{f}_1}L_{\mathbf{f}_1}\mathbf{g}$, $L_{\mathbf{f}_0}L_{\mathbf{f}_0}L_{\mathbf{f}_0}\mathbf{g}$, $L_{\mathbf{f}_0}L_{\mathbf{f}_0}L_{\mathbf{f}_1}\mathbf{g}$, $L_{\mathbf{f}_0}L_{\mathbf{f}_0}L_{\mathbf{f}_1}\mathbf{g}$, $L_{\mathbf{f}_0}L_{\mathbf{f}_1}L_{\mathbf{f}_0}\mathbf{g}$, $L_{\mathbf{f}_0}L_{\mathbf{f}_1}L_{\mathbf{f}_1}\mathbf{g}$, $L_{\mathbf{f}_1}L_{\mathbf{f}_0}L_{\mathbf{f}_0}\mathbf{g}$, $L_{\mathbf{f}_1}L_{\mathbf{f}_0}L_{\mathbf{f}_1}\mathbf{g}$, $L_{\mathbf{f}_1}L_{\mathbf{f}_1}L_{\mathbf{f}_0}\mathbf{g}$, $L_{\mathbf{f}_1}L_{\mathbf{f}_1}L_{\mathbf{f}_1}\mathbf{g}$, $L_{\mathbf{f}_0}L_{\mathbf{f}_0}L_{\mathbf{f}_0}L_{\mathbf{f}_0}\mathbf{g}$, $L_{\mathbf{f}_0}L_{\mathbf{f}_0}L_{\mathbf{f}_0}\mathbf{f}_1\mathbf{g}$, $L_{\mathbf{f}_0}L_{\mathbf{f}_0}L_{\mathbf{f}_1}\mathbf{f}_0\mathbf{g}$, $L_{\mathbf{f}_0}L_{\mathbf{f}_0}L_{\mathbf{f}_1}\mathbf{f}_1\mathbf{g}$,
$L_{\mathbf{f}_0}L_{\mathbf{f}_1}L_{\mathbf{f}_0}\mathbf{f}_0\mathbf{g}$.

The mathematical expressions for these coefficients rapidly become exceedingly complex, thus, and for illustrative purposes, only some coefficients are shown here:

$$L_{f_0}g_1 = \sum_{j=1}^{15} f_{0j}\frac{\partial g_1}{\partial x_j}(t=0) = i_1 k_v \mathrm{NF}\kappa\mathrm{B}_0 - a_1 \mathrm{I}\kappa\mathrm{B}\alpha_{n0}\mathrm{NF}\kappa\mathrm{B}_{n0}$$

$$L_{f_0}g_2 = \sum_{j=1}^{15} f_{0j}\frac{\partial g_2}{\partial x_j}(t=0) = -c_{6a}NF - c_{5a}\mathrm{I}\kappa\mathrm{B}\alpha_0 - i_{1a}\mathrm{I}\kappa\mathrm{B}\alpha_0 + e_{1a}\mathrm{I}\kappa\mathrm{B}\alpha_{n0} + c_{4a}\mathrm{I}\kappa\mathrm{B}\alpha_{t0} + e_{2a}(\mathrm{I}\kappa\mathrm{B}\alpha_n|\mathrm{NF}\kappa\mathrm{B}_n)_0$$

$$\vdots$$

$$L_{f_1}g_1 = \sum_{j=1}^{15} f_{1j}\frac{\partial g_1}{\partial x_j}(t=0) = 0$$

$$\vdots$$

$$L_{f_1}g_5 = \sum_{j=1}^{15} f_{1j}\frac{\partial g_5}{\partial x_j}(t=0) = k_1\mathrm{IKKn}_0$$

$$\vdots$$

$$L_{\mathbf{f}_0}L_{\mathbf{f}_0}g_1 = \sum_{j=1}^{15} f_{0j}\frac{\partial L_{\mathbf{f}_0}g_1}{\partial x_j}(t=0) = i_1k_v(c_{6a}NF - i_1\mathrm{NF}\kappa\mathrm{B}_0 - \mathrm{I}\kappa\mathrm{B}\alpha_0\mathrm{NF}\kappa\mathrm{B}_0) - a_1\mathrm{NF}\kappa\mathrm{B}_{n0}$$
$$(i_{1a}k_v\mathrm{I}\kappa\mathrm{B}\alpha_0 - e_{1a}k_v\mathrm{I}\kappa\mathrm{B}\alpha_{n0} - a_1\mathrm{I}\kappa\mathrm{B}\alpha_{n0}\mathrm{NF}\kappa\mathrm{B}_{n0}) - a_1\mathrm{I}\kappa\mathrm{B}\alpha_{n0}(i_1k_v\mathrm{NF}\kappa\mathrm{B}_0 - a_1\mathrm{I}\kappa\mathrm{B}\alpha_{n0}\mathrm{NF}\kappa\mathrm{B}_{n0})$$

$$\vdots$$

$$L_{\mathbf{f}_0}L_{\mathbf{f}_1}g_5 = \sum_{j=1}^{15} f_{0j}\frac{\partial L_{\mathbf{f}_1}g_5}{\partial x_j}(t=0) = k_1(k_{prod} - kdeg\mathrm{IKKn}_0)$$

$$\vdots$$

$$L_{\mathbf{f}_1}L_{\mathbf{f}_0}g_2 = \sum_{j=1}^{15} f_{1j}\frac{\partial L_{\mathbf{f}_0}g_2}{\partial x_j}(t=0) = k_1\mathrm{IKKn}_0(-a_3NF - a_2\mathrm{I}\kappa\mathrm{B}\alpha_0)$$

$$L_{\mathbf{f}_1}L_{\mathbf{f}_0}g_3 = \sum_{j=1}^{15} f_{1j}\frac{\partial L_{\mathbf{f}_0}g_3}{\partial x_j}(t=0) = 0$$

$$L_{\mathbf{f}_1}L_{\mathbf{f}_0}g_4 = \sum_{j=1}^{15} f_{1j}\frac{\partial L_{\mathbf{f}_0}g_4}{\partial x_j}(t=0) = k_1k_{deg}\mathrm{IKKn}_0 + k_1\mathrm{IKKn}_0(-kdeg - a_3NF - a_2\mathrm{I}\kappa\mathrm{B}\alpha_{n0})$$

$$\vdots$$

Figure 1 presents the corresponding tableau.

In the following steps the minimum and reduced tableaus are sought and the parameters are iteratively computed as functions of the generating series coefficients. Figure 2 presents the minimum and subsequent tableaus. From the figure it is clear that in the first step it is possible to directly compute c_{3a}, k_1 and i_1; in the next iteration k_2 and i_{1a} can be also be directly computed; two systems of two equations are then used to compute c_{4a}, e_{2a}, k_{prod} and k_{deg}. Remaining equations are used to compute t_1, t_2, c_5 and k_3. Solution of the system of equations is unique thus the model is said to be identifiable (for the parameters $\boldsymbol{\theta}$).

	t_1	t_2	c_{3a}	c_{4a}	c_5	k_1	k_2	k_3	k_{prod}	k_{deg}	i_1	e_{2a}	i_{1a}
S_1^{0}											x		
S_2^{0}				x								x	x
S_4^{0}									x	x			
S_6^{0}			x										
S_5^{1}						x							
S_1^{00}											x		x
S_2^{00}			x	x								x	x
S_3^{00}											x		
S_4^{00}									x	x			
S_6^{00}			x								x		
S_5^{01}						x			x	x			
S_5^{11}						x	x						
S_1^{000}				x							x	x	x
S_2^{000}			x	x							x	x	x
S_3^{000}											x		x
S_4^{000}									x	x			
S_6^{000}			x								x		
S_5^{001}						x			x	x			
S_2^{010}				x		x			x	x		x	x
S_4^{010}				x		x			x	x		x	x
S_5^{010}				x		x		x	x	x		x	x
S_5^{011}					x	x	x		x	x			
S_2^{100}				x		x		x		x		x	x
S_4^{100}	x	x		x		x		x		x		x	x
S_5^{100}	x	x		x		x		x		x		x	x
S_5^{101}					x	x	x			x			
S_2^{110}						x	x						
S_4^{110}						x	x	x		x			
S_5^{110}						x	x			x			
S_5^{111}						x	x						
S_1^{0000}			x	x							x	x	x
S_2^{0000}			x	x							x	x	x
S_3^{0000}				x							x	x	x
S_4^{0000}									x	x			
S_6^{0000}			x	x							x	x	x
S_5^{0001}						x			x	x			
S_2^{0010}			x	x		x			x	x	x	x	x
S_4^{0010}			x	x		x		x	x	x	x	x	x
S_5^{0010}			x	x		x			x	x	x	x	x
S_5^{0011}					x	x	x		x	x			
S_2^{0100}			x	x		x			x	x	x	x	x
S_4^{0100}	x	x	x	x		x		x	x	x	x	x	x
S_5^{0100}	x	x	x	x					x	x	x	x	x

Figure 1: *Schematic representation of the complete identifiability tableau.* s_j^I regards the coefficient corresponding to the successive Lie derivatives of indices I of g_j, for example, $s_1^{001} = L_{\mathbf{f}_0} L_{\mathbf{f}_0} L_{\mathbf{f}_1} g_1$. A cross in the coordinates (i, j) indicates that the corresponding non-zero generating series coefficient depends on the parameter θ_j.

Minimum tableau

	t_1	t_2	c_{3a}	c_{4a}	c_5	k_1	k_2	k_3	k_{prod}	k_{deg}	i_1	e_{2a}	i_{1a}
S_1^0											(x)		
S_2^0				x								x	x
S_4^0									x	x			
S_6^0			(x)										
S_5^1						(x)							
S_1^{00}											x		x
S_4^{00}									x	x			
S_5^{11}						x	x						
S_1^{000}				x							x	x	x
S_5^{010}				x		x		x	x	x		x	x
S_5^{011}					x	x	x		x	x			
S_4^{100}	x	x		x		x		x		x		x	x
S_4^{0100}	x	x	x	x		x		x	x	x	x	x	x

1st reduced tableau

	t_1	t_2	c_{4a}	c_5	k_2	k_3	k_{prod}	k_{deg}	e_{2a}	i_{1a}
S_2^0			x						x	x
S_4^0							x	x		
S_1^{00}										(x)
S_4^{00}							x	x		
S_5^{11}					(x)					
S_1^{000}			x						x	x
S_5^{010}			x			x	x	x	x	x
S_5^{011}				x	x		x	x		
S_4^{100}	x	x	x			x		x	x	x
S_4^{0100}	x	x	x			x	x	x	x	x

2nd reduced tableau

	t_1	t_2	c_{4a}	c_5	k_3	k_{prod}	k_{deg}	e_{2a}
S_2^0			x					x
S_4^0						x	x	
S_4^{00}						x	x	
S_1^{000}			x					x
S_5^{010}			x		x	x	x	x
S_5^{011}				x		x	x	
S_4^{100}	x	x	x		x		x	x
S_4^{0100}	x	x	x		x	x	x	x

3rd reduced tableau

	t_1	t_2	c_5	k_3
S_5^{010}				(x)
S_5^{011}			(x)	
S_4^{100}	x	x		x
S_4^{0100}	x	x		x

Figure 2: *Minimum and reduced tableaus.* Green crosses represent those parameters that can be computed from a single equation of the system. Green circles correspond to those parameters that may be uniquely identified, i.e. only one solution exist. Red crosses represent possible identifiability problems, i.e. sets of parameters that require more than 2 equations to be identified if possible. Red boxes and arrows represent sets of equations that result in an unique solution for the parameters.

Global ranking of parameters

As described in the main text there are several indexes that may be used to rank the parameters in order of their relative influence on model predictions. In practice the δ_{msqr} is probably the most widely used. However different criteria may lead to different results, thus results obtained with all criteria are shown in Figure s3. From the figure it is clear that all criteria lead to the same conclusions even though the relative order of the most relevant parameters may slightly vary from one criterion to the other.

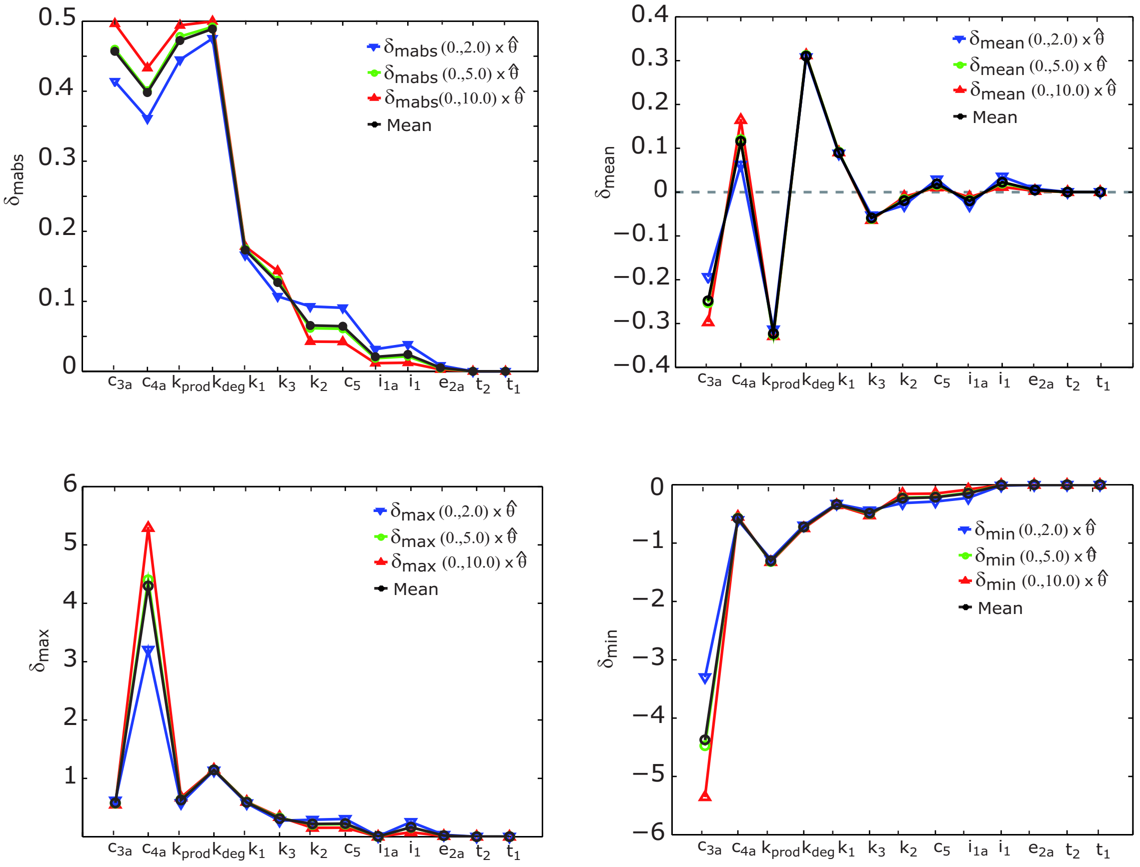

Figure 3: *Global ranking of parameters.* Four figures correspond to different ranking criteria, i.e. δ_{mabs}, δ_{mean}, δ_{max} and δ_{min}. Values are ordered using the mean δ_{msqr} as reference.

Optimal experimental design and corresponding identifiability analysis

The following tables present the detailed results after the optimal design of experiments.

Parameter	$\boldsymbol{\theta}^*$	$\boldsymbol{\mu}^{ES2}$	$\delta^{ES2}(in\%)$	C_{θ}^{ES2}	RC_{θ}^{ES2} (in %)
c_5	3.00×10^{-4}	3.01×10^{-4}	0.38	9.72×10^{-5}	32.3
k_1	2.50×10^{-3}	2.50×10^{-3}	0.19	4.49×10^{-4}	18.0
k_2	0.10	0.10	0.51	0.01	14.9
k_3	1.50×10^{-3}	1.50×10^{-3}	0.10	8.20×10^{-5}	5.48
k_{prod}	2.50×10^{-5}	2.51×10^{-5}	0.42	5.97×10^{-6}	23.8
k_{deg}	1.25×10^{-4}	1.25×10^{-4}	0.44	3.33×10^{-5}	26.5
i_1	2.50×10^{-3}	2.50×10^{-3}	0.12	2.60×10^{-4}	10.4
i_{1a}	1.00×10^{-3}	1.00×10^{-3}	0.40	8.98×10^{-5}	8.94

Table 1: Practical identifiability analysis for the experimental scheme *ES2* with $(0\hat{\theta}_i, 10\hat{\theta}_i)$ represents the nominal value for the parameters; $\boldsymbol{\mu}^{ES2}$ is the parameter mean value computed by the Monte-Carlo based approach; δ^{ES2} is the relative distance between the mean and the nominal computed as $\delta^{REF} = 100\frac{|\boldsymbol{\theta}^* - \boldsymbol{\mu}^{ES2}|}{\boldsymbol{\theta}^*}$, C_{θ}^{ES2} corresponds to the predicted maximum uncertainty of the given parameter and RC_{θ}^{ES2} represents the uncertainty with respect to $\boldsymbol{\mu}^{ES2}$ in %.

Parameter	$\boldsymbol{\theta}^*$	$\boldsymbol{\mu}^{ES3}$	$\delta^{ES3}(in\%)$	C_{θ}^{ES3}	RC_{θ}^{ES3} (in %)
c_5	3.00×10^{-4}	3.01×10^{-4}	0.60	5.09×10^{-5}	16.9
k_1	2.50×10^{-3}	2.50×10^{-3}	0.18	2.67×10^{-4}	10.7
k_2	0.10	0.10	0.25	0.008	7.85
k_{prod}	2.50×10^{-5}	2.50×10^{-5}	0.05	3.29×10^{-6}	13.2
k_{deg}	1.25×10^{-4}	1.25×10^{-4}	0.03	1.95×10^{-5}	15.6

Table 2: Practical identifiability analysis for the experimental scheme *ES3* with $(0\hat{\theta}_i, 10\hat{\theta}_i)$ represents the nominal value for the parameters; $\boldsymbol{\mu}^{ES3}$ is the parameter mean value computed by the Monte-Carlo based approach; δ^{ES3} is the relative distance between the mean and the nominal computed as $\delta^{REF} = 100\frac{|\boldsymbol{\theta}^* - \boldsymbol{\mu}^{ES3}|}{\boldsymbol{\theta}^*}$, C_{θ}^{ES3} corresponds to the predicted maximum uncertainty of the given parameter and RC_{θ}^{ES3} represents the uncertainty with respect to $\boldsymbol{\mu}^{ES3}$ in %.

The following figures detail the evolution of the expected uncertainty for every parameters throughout the identification procedure.

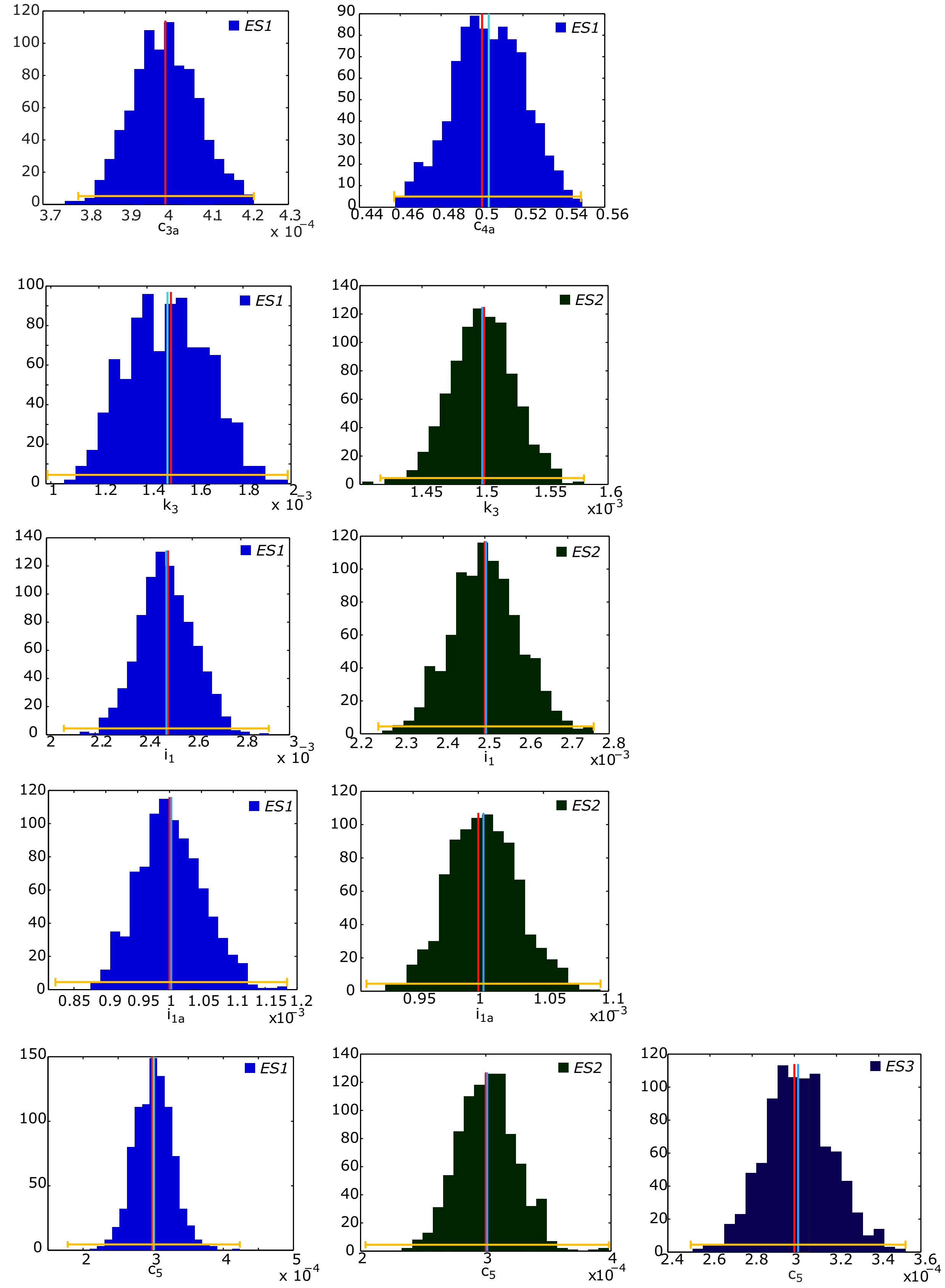

Figure 4: *Evolution of the expected uncertainty.* Red line indicates the nominal value of the parameter, blue line indicates the mean value for the given experiment and yellow line indicates the estimated expected uncertainty.

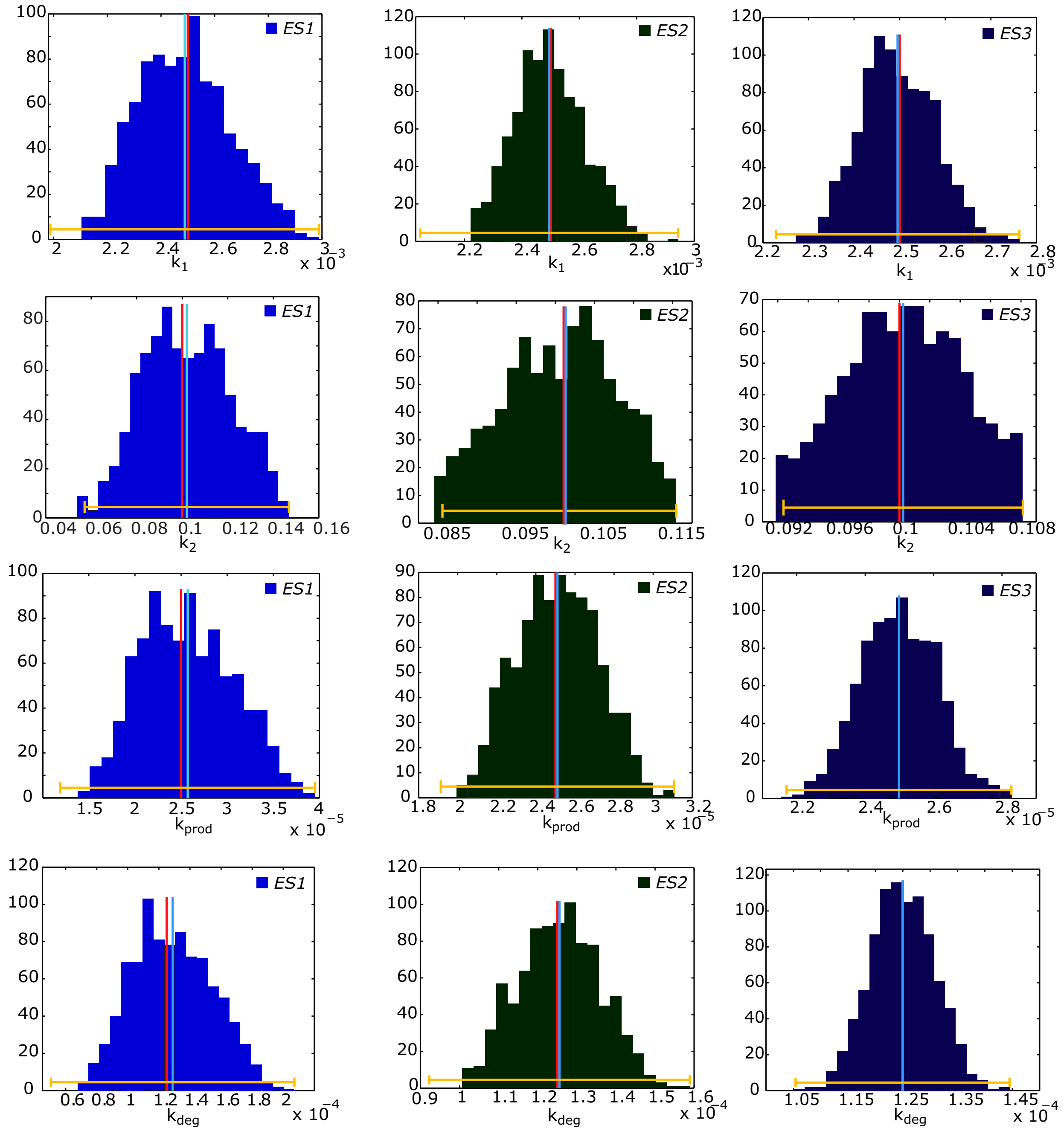

Figure 5: *Evolution of the expected uncertainty.* Red line indicates the nominal value of the parameter, blue line indicates the mean value for the given experiment and yellow line indicates the estimated expected uncertainty.
